# Texture-controlled growth of large-scale single-crystal metal foils

**DOI:** 10.1093/nsr/nwaf360

**Published:** 2025-08-28

**Authors:** Yu Wang, Zhibin Zhang, Yilin Jia, Min Ding, Chong Zhao, Qinghe Wang, Qile Wu, Wanting Sun, Zhi Huang, Menglin He, Zhiqiang Ding, Wei Liang, Kun Zhou, Mengze Zhao, Jijun Wang, Run Zhang, Xiangbin Yue, Minqiang Gao, Renguo Guan, Lin Zhou, Enge Wang, Ying Fu, Muhong Wu

**Affiliations:** Songshan Lake Materials Laboratory, China; State Key Laboratory for Mesoscopic Physics, Frontiers Science Centre for Nano-optoelectronics, School of Physics, Peking University, China; Songshan Lake Materials Laboratory, China; Songshan Lake Materials Laboratory, China; Songshan Lake Materials Laboratory, China; State Key Laboratory for Mesoscopic Physics, Frontiers Science Centre for Nano-optoelectronics, School of Physics, Peking University, China; National Laboratory of Solid State Microstructures, College of Engineering and Applied Sciences, Nanjing University, China; Songshan Lake Materials Laboratory, China; Songshan Lake Materials Laboratory, China; Songshan Lake Materials Laboratory, China; Songshan Lake Materials Laboratory, China; Songshan Lake Materials Laboratory, China; Songshan Lake Materials Laboratory, China; State Key Laboratory for Mesoscopic Physics, Frontiers Science Centre for Nano-optoelectronics, School of Physics, Peking University, China; Songshan Lake Materials Laboratory, China; Songshan Lake Materials Laboratory, China; Songshan Lake Materials Laboratory, China; School of Materials Science and Engineering, Dalian Jiaotong University, China; School of Materials Science and Engineering, Dalian Jiaotong University, China; National Laboratory of Solid State Microstructures, College of Engineering and Applied Sciences, Nanjing University, China; International Centre for Quantum Materials, Collaborative Innovation Centre of Quantum Matter, Peking University, China; Tsientang Institute for Advanced Study, China; Songshan Lake Materials Laboratory, China; Songshan Lake Materials Laboratory, China; Institute of Atomic Manufacturing, International Institute for Interdisciplinary and Frontiers, Beihang University, China; Interdisciplinary Institute of Light-Element Quantum Materials and Research Centre for Light-Element Advanced Materials, Peking University, China

## Abstract

This study presents a texture-controlled strategy for fabricating single-crystal metal foils, enabling the high-quality large-scale production with broad material compatibility.

Single-crystal metal foils have emerged as critical materials in modern technologies, particularly for high-frequency communication systems, next-generation microelectronics and advanced energy conversion devices [1]. Achieving ultra-smooth surfaces and low defect densities in single crystals is essential to overcome persistent challenges such as electron scattering, thermal degradation and interfacial failures [2,3]. In recent years, several methods have been developed to realize large-scale single-crystal growth, including thermal gradient control, contact-free annealing and seeded growth techniques, which primarily focus on energy engineering (surface, interface and strain energy optimization) to promote grain nucleation and growth in commercial metal foils [4–7]. However, these approaches face fundamental compatibility limitations due to the structural heterogeneity of industrial feedstocks. Commercial foils typically undergo rolling deformation and recrystallization annealing during manufacturing, resulting in substantial residual strain energy and batch-dependent recrystallization textures [8]. The absence of standardized processing protocols results in pronounced variability in texture type across production batches. Such structural variability imposes a fundamental bottleneck for the reliable mass production of single-crystal foils. Despite significant progress in understanding texture components and formation mechanisms, quantitative insights into the control of texture development and its correlation with single-crystal evolution remain limited [9,10]. Notably, although a pioneering demonstration of meter-scale single-crystal foils in 2017 [4], the field has struggled to scale up from laboratory success to industrial production. Bridging this scalability gap requires a comprehensive insight into the structural evolution and energy transformation pathways from polycrystalline feedstocks to single-crystal foils.

Here, we present a texture-controlled strategy for fabricating single-crystal metal foils, involving three key stages (Fig. 1a and Fig. S1a–c): (i) Cold rolling deformation, which processes raw materials into foils, creating a deformed microstructure with a high density of dislocations and introducing substantial stored energy into the foils. For raw face-centered cubic (FCC) metal material, plastic deformation predominantly occurs along the rolling and thickness directions, generating a high density of vacancies and dislocations that continuously accumulate stored energy; a specific dislocation alignment forms according to the preferential slip systems in the metal, inducing a characteristic biaxial stress distribution along the rolling and thickness directions (Fig. S1d). (ii) Low-temperature recrystallization annealing (around one-third of the melting point) [11], which eliminates dislocations, releases the stored energy and develops a uniform {100} texture across the foils due to their highest efficiency in releasing stored energy (Fig. S1e) [9,12]. (iii) High-temperature single-crystallization annealing (near the melting point), which promotes abnormal grain growth regulated by the texture, ultimately achieving the fabrication of large-scale single-crystal metal foils (Fig. S1f). The obtained facet can be tailored by the available energy modulation methods. Specifically, the surface energy-driven method yields a (111) facet (Fig. S2a), while the interface energy-driven method enables the formation of a high-index (*hkl*) facet (Fig. S2b). In both cases, the nucleus forms a stable orientation relationship with the {100} texture grains and develops into a large single crystal through abnormal grain growth.

**Figure 1.**
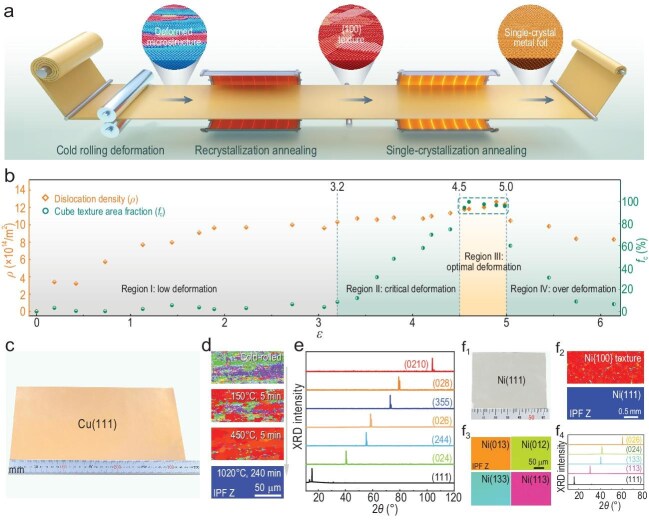
Texture-controlled strategy for fabricating single-crystal metal foils. (a) Overview of the designed process chain, including cold rolling, recrystallization annealing and single-crystallization annealing. (b) The guiding framework for single-crystal Cu foil fabrication, illustrating the evolution of geometrically necessary dislocation density (*ρ*) with true strain (*ε*) during the cold rolling process and the corresponding cube texture area fraction (*f*_c_) after recrystallization annealing. (c) Optical image of the synthesized single-crystal Cu(111) foil with the size of 33 × 20 cm^2^. (d) EBSD evolution of a cold-rolled Cu foil from Region III during recrystallization and the single-crystallization annealing process. (e) XRD 2*θ* scans (Ag-based target) of the fabricated single-crystal Cu foils. (f_1_) Optical image of the single-crystal Ni(111) foil with the size of 6 × 5 cm^2^. (f_2_) EBSD maps of the Ni{100} texture and the single-crystal Ni(111) foil. (f_3_) EBSD maps of four kinds of single-crystal Ni foils. (f_4_) XRD 2*θ* scans (Ag-based target) of the fabricated single-crystal Ni foils.

To validate the effectiveness of this approach, we first use copper (Cu) as a model metal, focusing on the interplay between strain (recorded by true strain [13], $\varepsilon = {\varepsilon }_0 + ln( {{h}_0/h} )$, where *ε*_0_ represents the initial strain, and *h*_0_ and *h* are the initial and final thickness, respectively), stored energy (quantified by geometrically necessary dislocation density, *ρ*), texture (characterized by cube texture fraction, *f*_c_), and their collective impact on single-crystal growth. To fully illustrate the transition from a low-energy (dislocation-free) state to a high-energy (dislocation-accumulated) state, a single-crystal Cu plate was used as the starting material (*ε*_0_ = 0, Fig. S3a–d, see Supplementary notes for more details).

The strain applied to the raw Cu plates can be categorized into four distinct regions for fabricating single crystals (Fig. 1b). In the low-deformation region (Region Ⅰ, where 0 < $\varepsilon $ < 3.2), dislocations rapidly accumulated, resulting in a sharp rise in ρ to about 10 × 10^14^ m^–2^ (Fig. 1b and Fig. S3e_1_). However, despite the rapid increase in dislocation density, the stored energy remained insufficient, leading to the formation of a weak {100} texture (${f}_{\mathrm{c}}$< 10%) after recrystallization annealing (Fig. S3e_2_). Following the single-crystallization annealing, the grains remained confined to the micrometer-to-millimeter size range, which rendered this region unsuitable for single-crystal fabrication (Fig. S3e_3_ and Fig. S4).

As the strain increased, it entered the critical deformation region (Region II, 3.2 < $\varepsilon $ < 4.5), where the dislocation density steadily rose to 10 × 10^14^ < $\rho $ < 11 × 10^14^ m^–2^ (Fig. 1b and Fig. S3f_1_). This facilitated a gradual enhancement of the {100} texture fraction, with *f*_c_ increasing to approximately 20% to 80% after recrystallization annealing (Fig. S3f_2_). During single-crystallization annealing, the moderate {100} texture facilitated significant grain growth, resulting in millimeter-scale grains in the Cu foils (Fig. S3f_3_ and Fig. S4).

Further increasing the deformation reached the optimal deformation region (Region III, 4.5 < $\varepsilon $ < 5.0), where the dislocation density peaked at 11 × 10^14^ < $\rho $ < 13 × 10^14^ m^–2^, indicating efficient accumulation of substantial stored energy (Fig. 1b and Fig. S3g_1_). In this region, a strong and uniform {100} texture with *f*_c_ of >90% developed after the recrystallization annealing (Fig. S3g_2_). With the subsequent single-crystallization annealing, this optimized texture enabled the fabrication of large-scale single-crystal Cu foils (Fig. S3g_3_ and Fig. S4).

It was noteworthy that an over-deformation region (Region IV, *ε* > 5.0) was identified. Due to the excessive stress concentrations, dynamic recrystallization occurred in localized areas with high stored energy, significantly reducing the dislocation density (Fig. 1b and Fig. S3h_1_) [14]. This resulted in the formation of fine recrystallized grains stabilized by high-angle grain boundaries. Consequently, the growth of {100} texture grains was hindered during recrystallization annealing, preventing the development of a strong {100} texture (Fig. S3h_2_). As a result, even after high-temperature single-crystallization annealing, this region only produced a fine-grain structure with limited grain growth (Fig. S3h_3_ and Fig. S4).

Building upon the investigation of cast single-crystal Cu plates, the established correlation among strain, stored energy and recrystallization texture reveals that sufficient stored energy and a well-defined {100} texture are critical prerequisites for the formation of high-quality single-crystal Cu foils. This rule was further validated in rolled and electrodeposited Cu plates, which displayed consistent trends in dislocation density evolution during cold rolling and in {100} texture development upon recrystallization annealing (Figs S5 and S6). Optimal true strains ε of approximately 4.8 and 4.5 for the rolled and electrodeposited plates, respectively, were found to enable the formation of nearly 100% {100} texture, and thereby the fabrication of large-area single-crystal Cu foils. To validate this criterion, we examined a series of commercial Cu foils with varying initial {100} texture fractions. We found that high-quality single crystals can be consistently obtained only when the {100} texture fraction approaches 100% (Fig. S7), highlighting the decisive role of initial uniform texture in governing the single-crystal fabrication.

Once a stable and uniform {100} texture is achieved in the optimized deformation region (Region III), single-crystal Cu foils can be readily fabricated using previously established methods. Initially, we employed a surface energy-driven method, which facilitated the transformation of the {100} texture into a single-crystal Cu(111) foil with the lowest surface energy. By utilizing a large furnace, the size of the single-crystal Cu(111) foil was extended to 33 × 20 cm² (Fig. 1c). The quality of this A4-size single crystal was confirmed via X-ray diffraction (XRD) 2*θ* and *φ* scan, high-angle annular dark-field scanning transmission electron microscopy (HAADF-STEM), low-energy electron diffraction (LEED) (Fig. S8), atomic force microscopy (AFM) (Fig. S9) and optical characterizations (Fig. S10), all verifying its high crystallinity and superior properties. The crystallization process was also monitored through electron backscatter diffraction (EBSD) (Fig. 1d), where it initiated at 150°C, with the {100} texture area fraction exceeding 60%, then expanded significantly at 450°C to over 90%. It finally reached about 100% at 950°C (see *in situ* evolution in Movies S1 and S2), upon which single-crystal growth was realized at 1020°C.

Additionally, we applied an interface energy-driven method to produce single-crystal Cu foils with high-index facets [6]. Following the seeded growth strategy, the {100}-textured Cu foils were oxidized to form a stable oxide layer (Fig. S11). Thus, the interface energy between the foil and the oxide, rather than surface energy, became the key driving force for the abnormal grain growth. As a result, high-index nuclei had the chance to form, and six kinds of single-crystal Cu foils with high-index facets were finally obtained (Fig. 1e and Fig. S12).

The similarity in the plastic deformation and recrystallization mechanisms between metals provides a robust theoretical foundation for the universality of our texture-controlled strategies. We further extended this strategy to nickel (Ni) and successfully fabricated centimeter-scale single-crystal Ni foils (Fig. 1f_1_). With optimized Ni foils with {100} texture fractions exceeding 90% (Fig. 1f_2_), we successfully synthesized 6 × 5 cm^2^ single-crystal Ni(111) foils through the surface energy-driven abnormal grain growth method (Fig. S13). Similarly, four kinds of high-index single-crystal Ni foils were also produced through the interface energy-driven abnormal grain growth method (Fig. S11), with their crystalline orientations confirmed using EBSD and XRD (Fig. 1f_3_ and f_4_).

In summary, we propose a texture-controlled strategy for the fabrication of high-quality single-crystal metal foils. The established comprehensive process chain encompasses rolling deformation to store energy, recrystallization annealing to develop a complete {100} texture, and abnormal grain growth treatment to enable the fabrication of single crystals. This strain-energy-texture regulation framework accommodates diverse initial states—including as-cast, rolled and electrodeposited—demonstrating broad adaptability. The demonstrated universality and scalability of this approach pave the way for their scalable industrial production and future potential applications.

## Supplementary Material

nwaf360_Supplemental_Files
