# Supplementary material for: Texture-controlled growth of large-scale single-crystal metal foils
Source: Natl Sci Rev. 2025 Aug 28;12(10):nwaf360. doi: 10.1093/nsr/nwaf360 (PMC12485617; doi:10.1093/nsr/nwaf360)
Supplement: nwaf360_Supplemental_Files [file nwaf360_supplemental_files.zip › Supplementary data-r.pdf]

Supporting Information

Texture-controlled growth of large-scale single-crystal metal foils

Yu Wang, Zhibin Zhang, Yilin Jia, Min Ding, *et al*.

Corresponding author: Muhong Wu, muhongwu@buaa.edu.cn;

Ying Fu, fuying@sslab.org.cn;

Enge Wang, egwang@pku.edu.cn;

Zhibin Zhang, zhibinzhang@pku.edu.cn

The PDF file includes:

Supplementary Notes

Figs S1 to S13

Other supporting information for this manuscript includes the following:

Movies S1 and S2

SUPPEMENTARY NOTES

Preparation of single-crystal Cu foils. The casting, rolled, and electrodeposited Cu plates (5 mm thickness, 99.9%) were purchased from Zhongke Crystal Materials Technology Co. Ltd. The casting plate with centimeter-scale grains was fabricated via directional solidification and further cut into single-crystal Cu plates. All Cu plates were cold rolled using a biaxial roller squeezer with a roll radius of 60 mm. The rolling process was controlled by adjusting the roll gap and maintaining at a fixed roller rotational speed of 50 Hz. The plates were gradually rolled to various thicknesses with a minimum of 10 μm. The total rolling reduction ratio was 98.9–99.3% (from 5 mm to ~35–55 μm), and the applied strain rate was estimated to range from 5–20 s^{-1} for the fabrication of single-crystal Cu foils. The cold-rolled Cu foils were placed on a quartz substrate and loaded into an atmospheric pressure chemical vapor deposition (CVD) furnace (Tianjin Kaiheng Co. Ltd. custom-designed). To produce recrystallized Cu foils, the furnace was heated to 450 °C in 45 min, then maintained at this temperature under a gas flow of 500 standard cubic centimeters per minute (sccm) Ar and 50 sccm H_2 for 2 h. Then for Cu(111) foils, the recrystallized foils were further heated to 1020–1060 °C in 60 min and held at this temperature for 3–6 h under the same gas flow. To produce Cu foils with high-index facets, the recrystallized foils were first heated to 150–650 °C in 10–60 min and then maintained at this temperature for 1–2 h in air to obtain an oxidized surface. Then the foils were heated to 1020–1060 °C in 1 h and maintained at this temperature under a gas flow of 500 sccm Ar and 50 sccm H_2 for 3–6 h.

Preparation of single-crystal Ni foils. A Ni plate (2 mm thickness, 99.6%, Zhongke Crystal Materials Technology Co. Ltd) was cold rolled to a final thickness of 20 μm, with a fixed roller rotational speed of 30 Hz. The total rolling reduction ratio was 97.5–99.0% (from 2 mm to ~20–50 μm), and the applied strain rate was estimated to range from 5–10 s^{-1} for the fabrication of single-crystal Ni foils. The rolled Ni foils were placed on an aluminum oxide plate and loaded into the CVD furnace. To produce recrystallized Ni foils, the furnace was heated to 500 °C in 50 min, then maintained at this temperature under a gas flow of 500 sccm Ar and 20 sccm H_2 for 2 h. To produce Ni(111) foils, the recrystallized foils were further heated to 1200–1400 °C in 2 h and held at this temperature for 3–6 h under the same gas flow. To produce Ni foils with high-index facets, the recrystallized foils were first heated to 150–650 °C in 10–60 min and then maintained at this temperature for 1–2 h in air to obtain an oxidized surface. Then the foils were heated to 1200–

1400 °C in 2 h and maintained at this temperature under a gas flow of 500 sccm Ar and 20 sccm H_2 for 3–6 h.

Characterizations. Optical images were obtained using an Olympus BX-51 fluorescence motorized microscope. EBSD characterizations were performed using an Oxford Instruments Aztec 2.0 system with an EBSD detector operating at 20 kV and a step size of 0.2 or 5 μm. The EBSD data were analysed using AztecCrystal, version 2.1 software. *In situ* EBSD measurements were performed using a Ciqtek SEM 5000X system, with the temperature increasing at 1 °C/min to 950 °C. Once the target temperature was reached, EBSD patterns were recorded with a step size of 0.3 μm. The area fraction (f_c) of the cube texture was calculated within a misorientation tolerance of 15°. HAADF-STEM images were obtained using a FEI Thermofisher Spectra 300, operated at 300 kV. XRD 2θ- and φ-scan measurements were conducted using a PANalytical Empyrean diffractometer equipped with Cu and silver (Ag) targets. The dielectric function was measured using an RC2 spectroscopic ellipsometer. Optical reflectance spectra were acquired in reflection mode using a CRAIC microspectrometer with unpolarized light incident perpendicularly to the sample surface. LEED patterns were obtained by a LEED-Auger spectrometers BDL800IR system in ultrahigh vacuum with a base pressure below 3×10^{-7} Pa. X-ray photoelectron spectroscopy (XPS) spectra were measured by a monochromatic Al Kα X-ray source using a Thermo Fisher ESCALAB XI+ electron energy analyser.

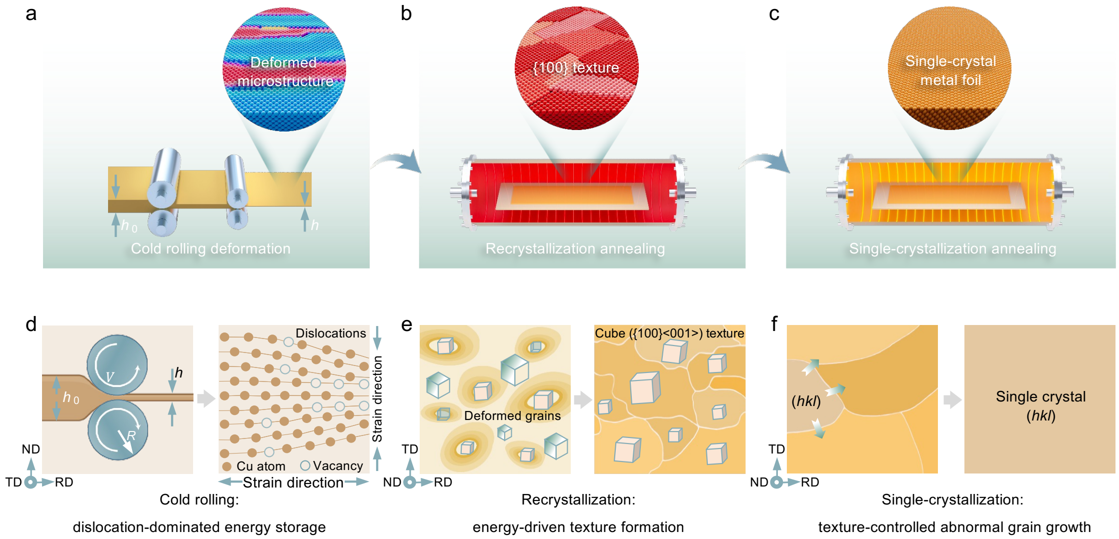

Fig. S1. Schematic of the single-crystal metal foil preparation process. (a–c) Overview of the cold rolling deformation (a), recrystallization annealing (b), and single-crystallization annealing (c) process for fabricating single-crystal metal foils. (d) Cold rolling process, where the metal thickness is reduced from h_0 to h, with stored energy accumulating through dislocation multiplication and alignment, generating a biaxial stress distribution along rolling and thickness directions. ND, RD, and TD represent the normal, rolling, and transverse directions, respectively. (e) Recrystallization annealing process, showing the transformation of deformed grains with large internal orientation gradients into a uniform cube ({100}<001>) texture. (f) Single-crystallization annealing process, where a large-scale single crystal is formed through texture-controlled abnormal grain growth, initiated by a (*hkl*)-oriented facet.

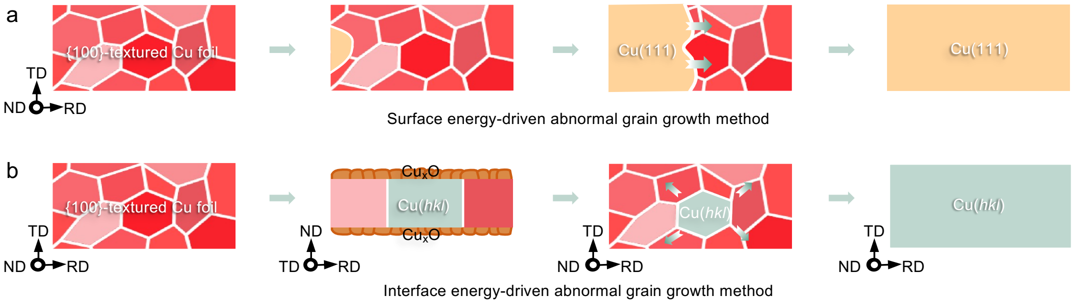

Fig. S2. Schematic of the single-crystal growth of metal foil. (a) Surface energy-driven abnormal grain growth method for achieving single crystals with (111) facet. (b) Interface energy-driven abnormal grain growth method for producing single crystals with high-index (hkl) facet.

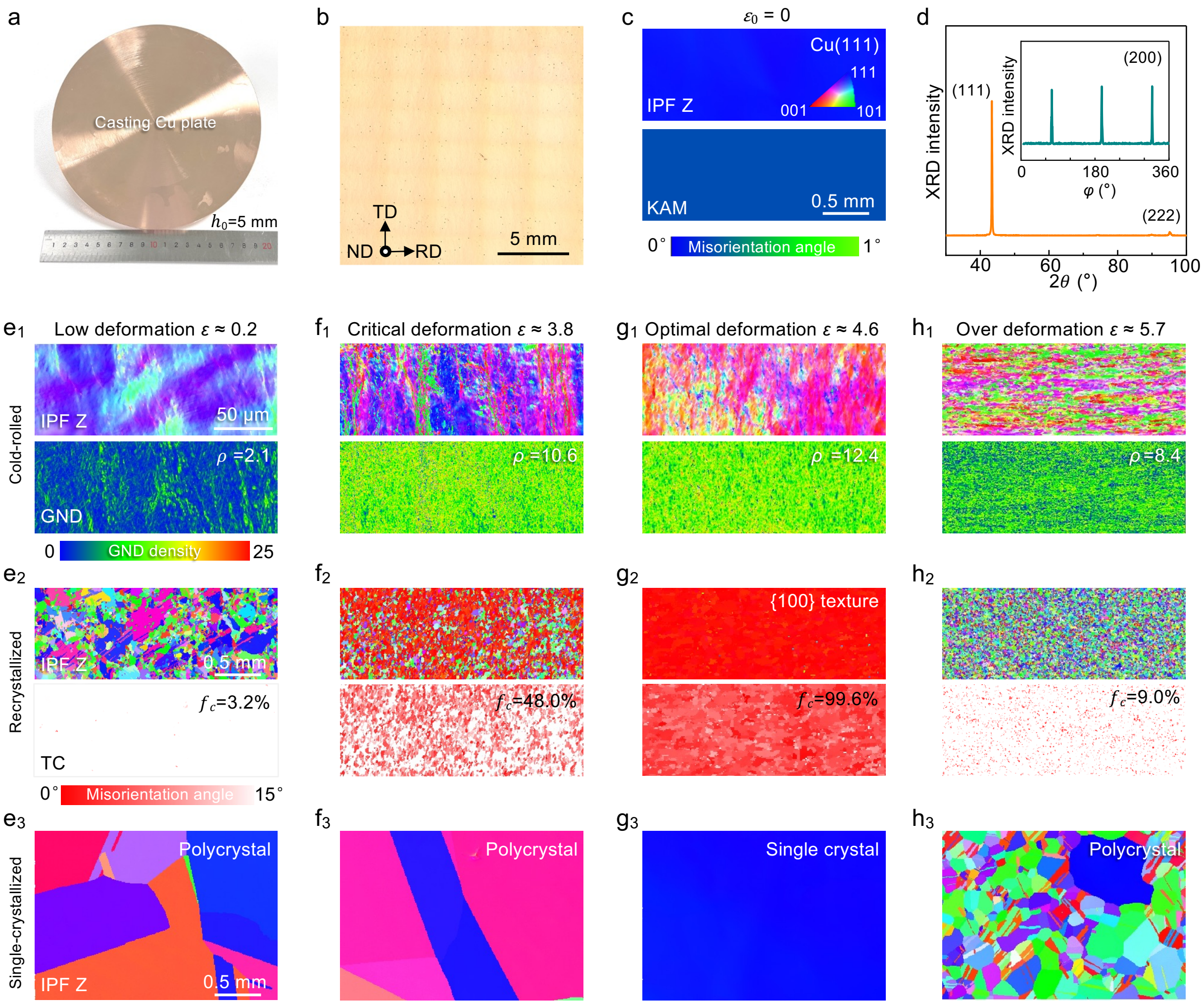

Fig. S3. Fabrication of single-crystal Cu foil from a casting Cu plate. (a–d) Optical image (a), metallographic image (b), EBSD maps (c), and XRD 2θ- and φ-scan (d, Cu-based target) of the Cu plate prepared by directional solidification casting, presenting centimeter-scale grains with identical crystallographic orientation. (e–h) EBSD maps of the Cu foils produced from the casting Cu plate in the cold-rolled, recrystallized, and single-crystallized states at varying strain levels. The results showed that the casting Cu plate required a strain of about $\varepsilon \approx 4.6$ to achieve nearly 100% cube texture and to form single crystals.

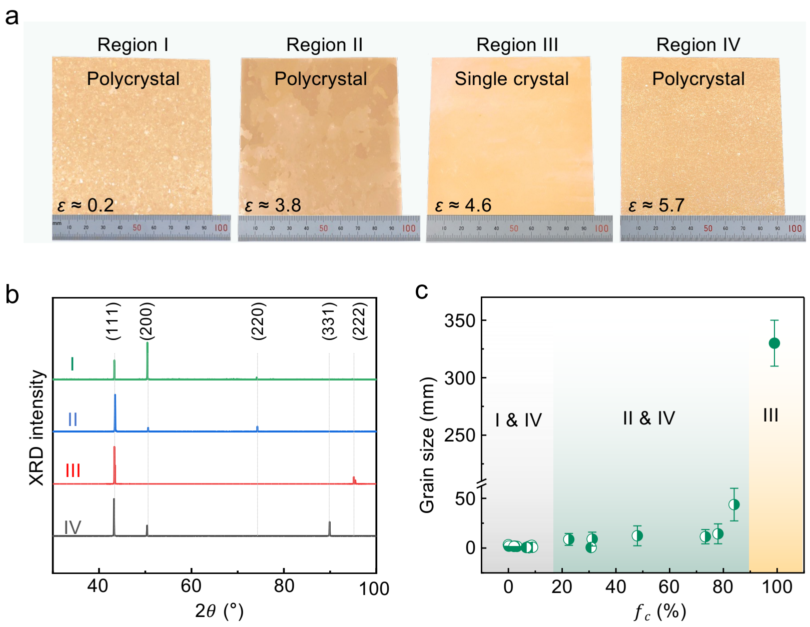

Fig. S4. (a) Optical images, (b) XRD 2θ-scans (Cu-based target), and (c) average grain sizes of the Cu foils after single-crystallization annealing from each deformation region, demonstrating that Region III (4.5 < ε < 5.0, 11×10^{14} < ρ < 13×10^{14} m^{-2} and f_c > 90%) is the optimal deformation range for fabricating single-crystal Cu foil.

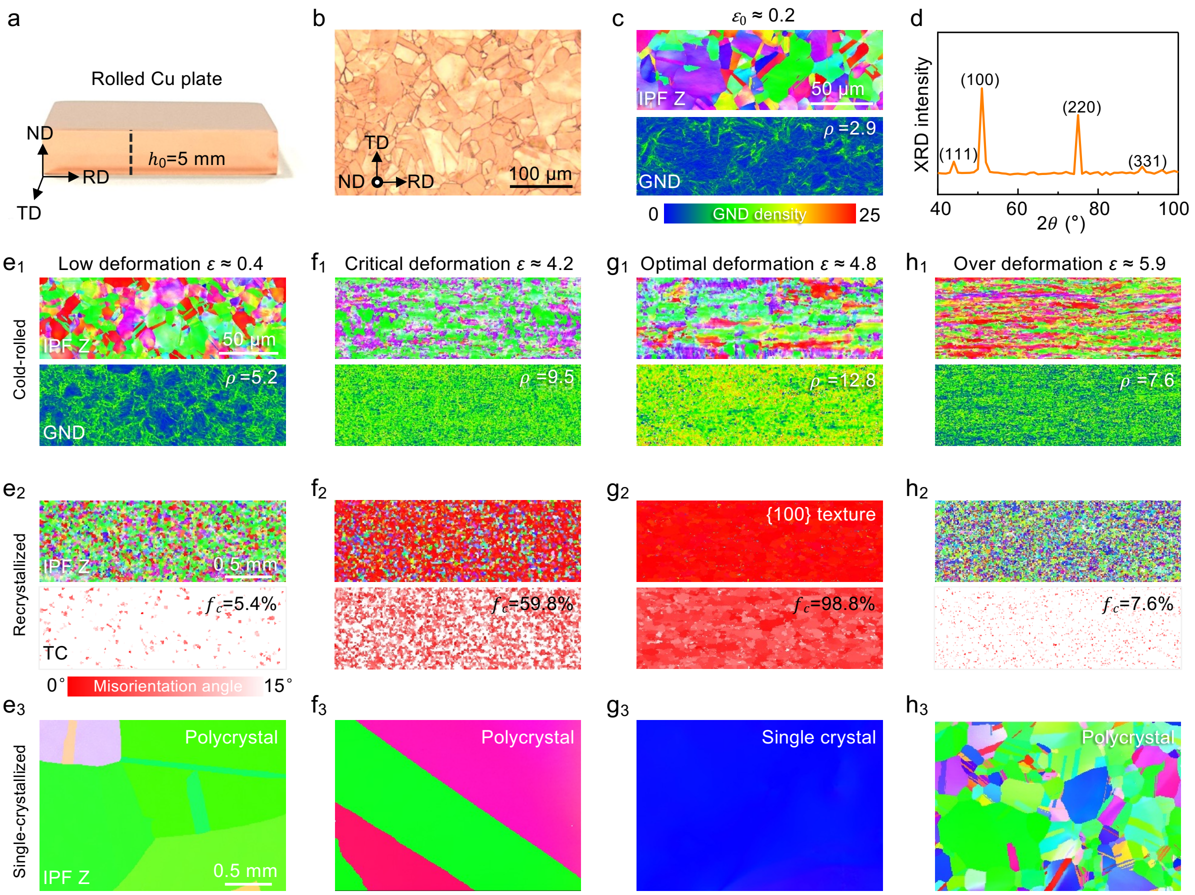

Fig. S5. Fabrication of single-crystal Cu foil from a rolled Cu plate. (a–d) Optical image (a), metallographic image (b), EBSD maps (c), and XRD 2θ-scan (d, Cu-based target) of the rolled Cu plate. (e–h) EBSD maps of the Cu foils produced from the rolled Cu plate in the cold-rolled, recrystallized, and single-crystallized states at varying strain levels. The results showed that the rolled Cu plate required a strain of about $\varepsilon \approx 4.8$ to achieve nearly 100% cube texture and to form single crystals.

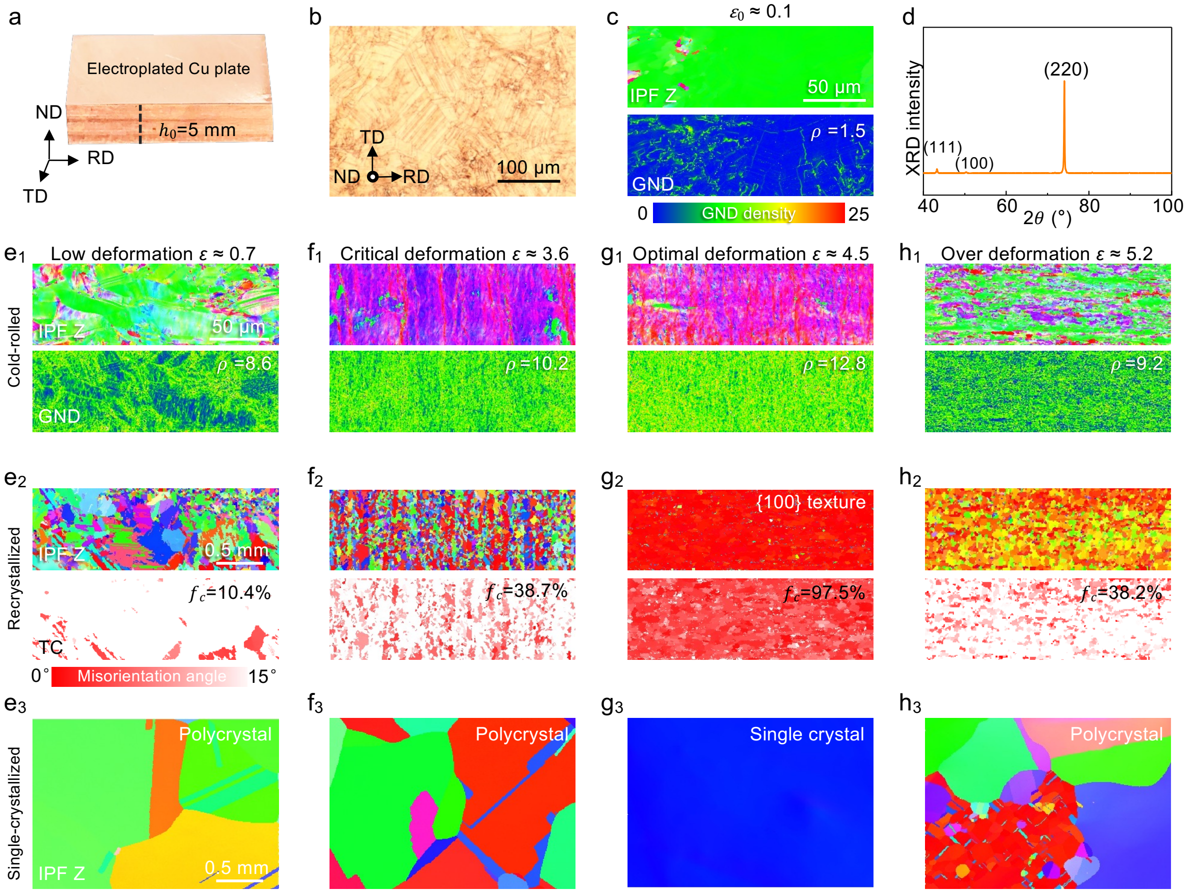

Fig. S6. Fabrication of single-crystal Cu foil from electrodeposited Cu plate. (a–d) Optical image (a), metallographic image (b), EBSD maps (c), and XRD 2θ-scan (d, Cu-based target) of the electrodeposited Cu plate. (e–h) EBSD maps the Cu foils produced from the electrodeposited Cu plate in the cold-rolled, recrystallized, and single-crystallized states at varying strain levels. The results showed that the electrodeposited Cu plate required a strain of about $\varepsilon \approx 4.5$ to achieve nearly 100% cube texture and to form single crystals.

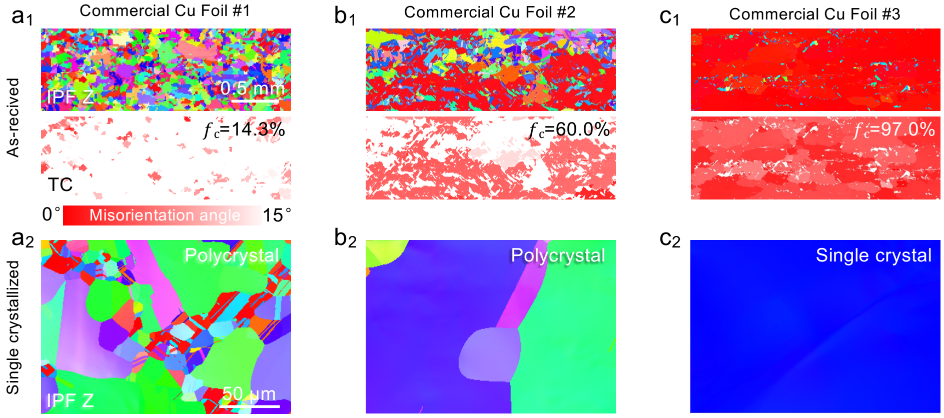

Fig. S7. The EBSD results of the as-received and single-crystallized commercial Cu foils with varying {100} texture fractions, which are f_c=14.3% (a_1 and a_2), f_c=60.0% (b_1 and b_2), and f_c=97.0% (c_1 and c_2).

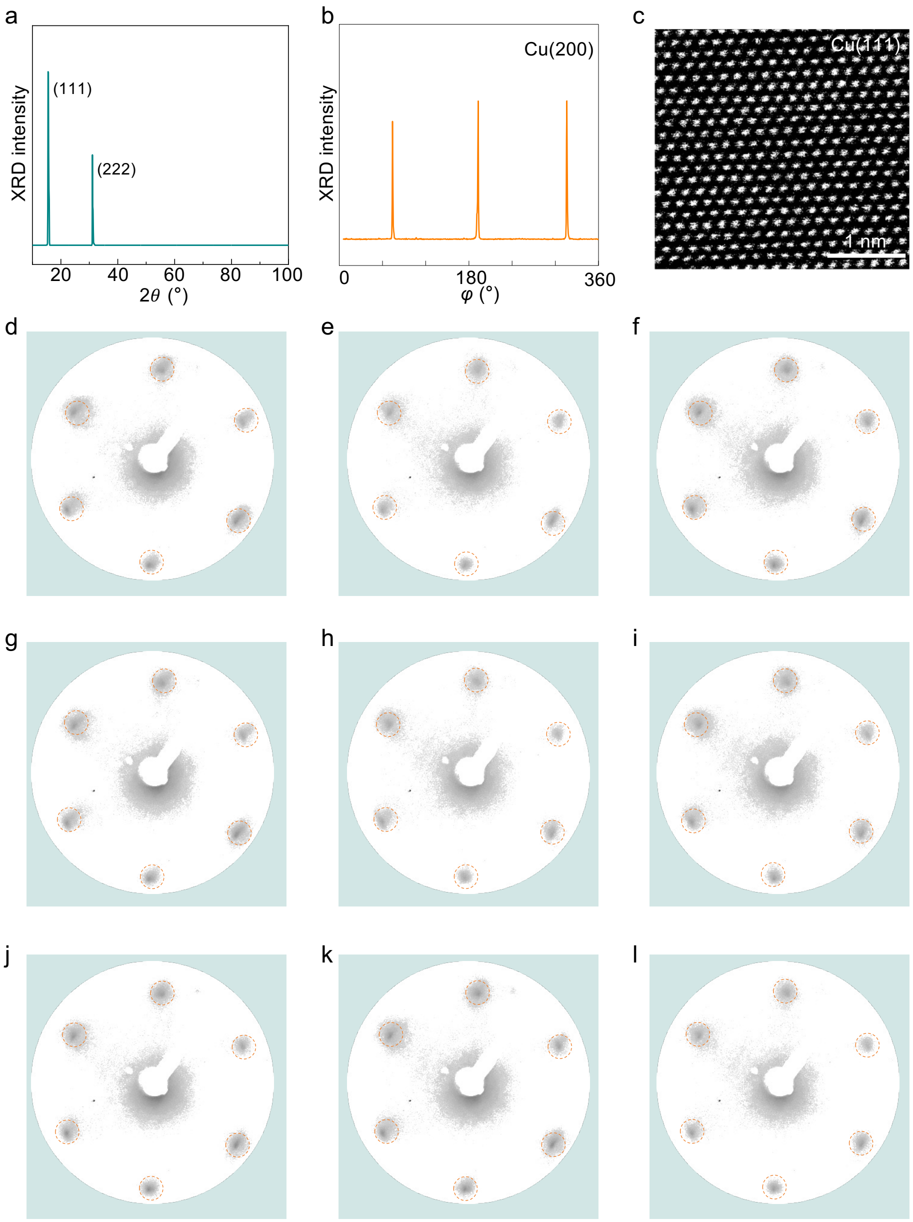

Fig. S8. Validation of the surface crystallinity and orientation consistency of the fabricated A4-size single-crystal Cu(111) foil. (a) The XRD 2θ-scan (Ag-based target) of the Cu(111) foil. (b) Azimuthal off-axis φ-scan showing three peaks corresponding to Cu(200). (c) HAADF-STEM image of the foil. (d–l) LEED patterns taken at 9 selected positions of the single-crystal Cu foil.

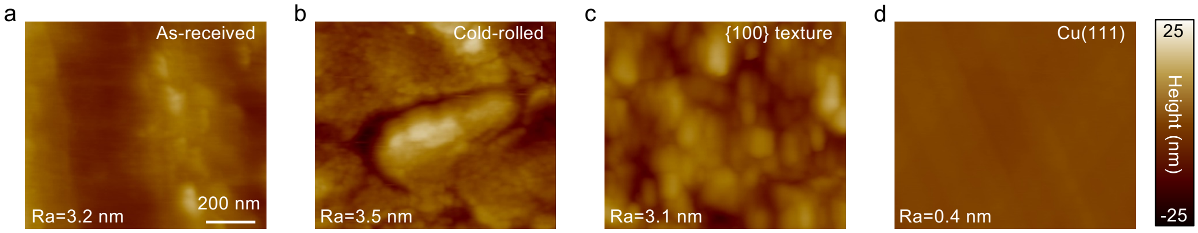

Fig. S9. AFM characterization of the surface morphology of Cu foils at different stages. AFM images of as-received raw Cu plate (a), cold-rolled Cu foil (b), recrystallized Cu foil with ~100% {100} texture (c), and single-crystal Cu(111) foil (d). All images are of the same size.

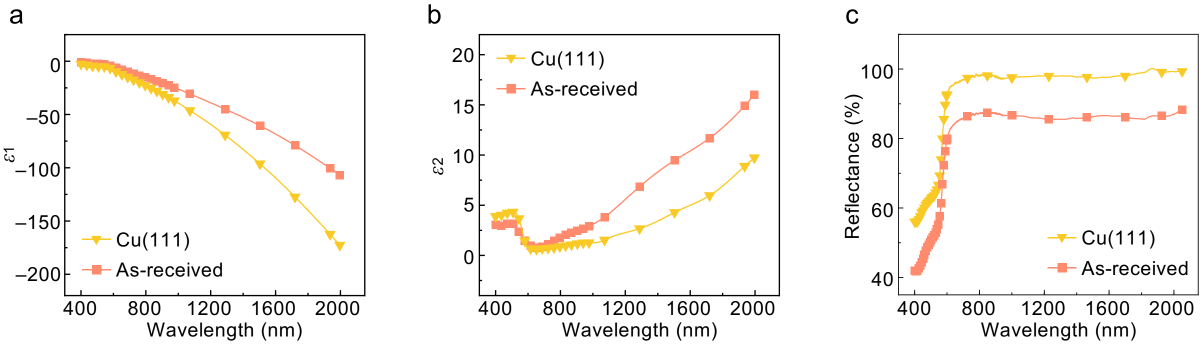

Fig. S10. Optical response comparison between polycrystalline and single-crystal Cu. Real ε_1 (a) and imaginary ε_2 (b) parts of permittivity, and reflectance spectra (c) of the as-received raw polycrystalline Cu plate and single-crystal Cu(111) foil. The single-crystal Cu(111) foil exhibited significantly lower optical loss and enhanced reflectivity across the measured spectral range.

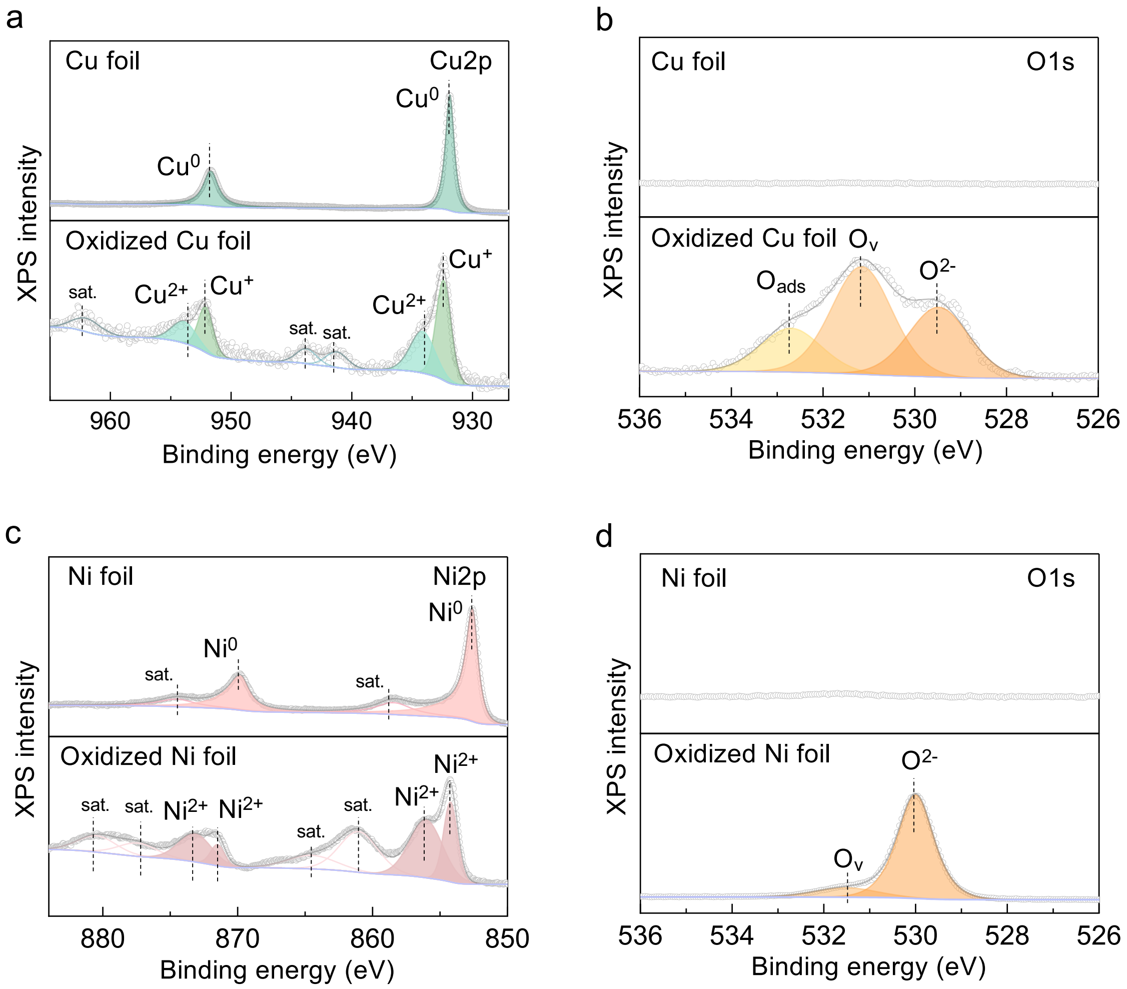

Fig. S11. XPS results of the Cu and Ni foils before and after the oxidation treatment. The presence of Cu^{+}, Cu^{2+}, and Ni^{2+} peaks confirmed successful oxidation of the metal foils, with O^{2-} peaks indicating oxidized valence states. Sat. denotes the satellite peak, O_v refers to chemisorbed oxygen, and O_{ads} indicates adsorbate oxygen.

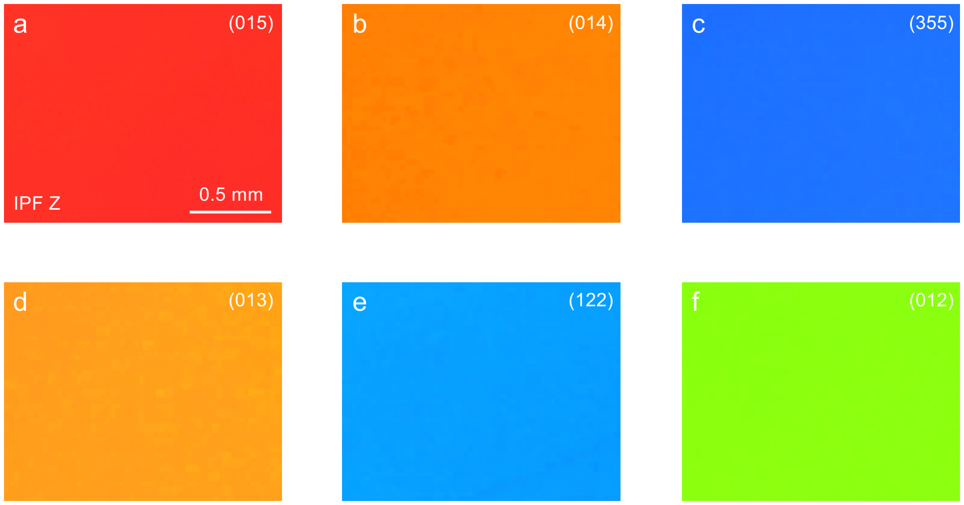

Fig. S12. EBSD-IPF Z maps of the six kinds of high-index single-crystal Cu foils.

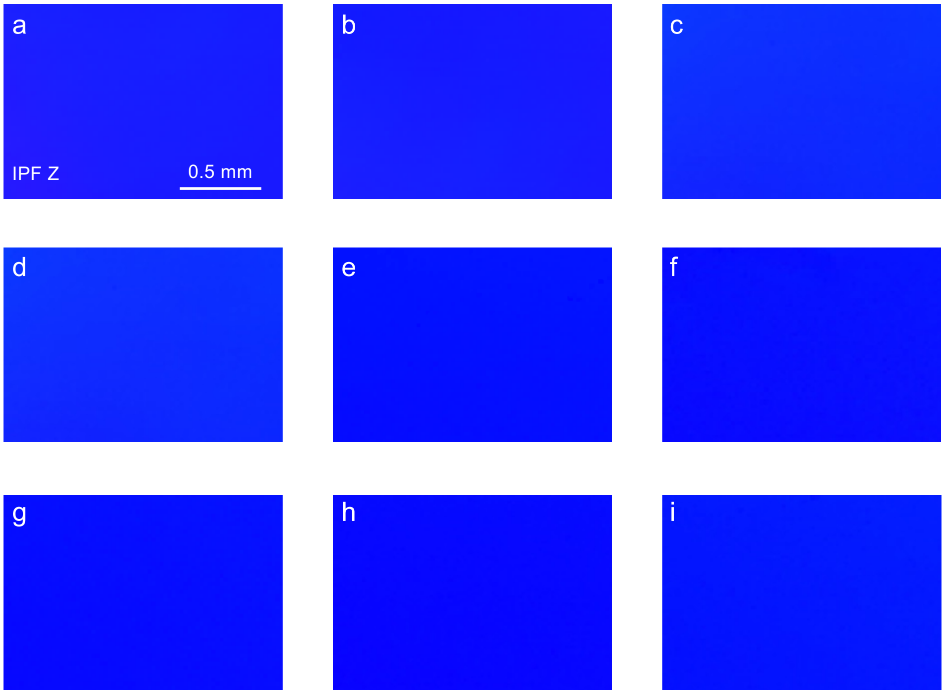

Fig. S13. EBSD-IPF Z maps taken at 9 selected positions across the single-crystal Ni(111) foil, confirming its uniform crystallographic orientation.
